# Metabolic Effects of Recombinant Human Growth Hormone Replacement Therapy on Juvenile Patients after Craniopharyngioma Resection

**DOI:** 10.1155/2022/7154907

**Published:** 2022-07-06

**Authors:** Shuying Li, Xi Wang, Yaling Zhao, Min Nie, Wen Ji, Jiangfeng Mao, Xueyan Wu

**Affiliations:** ^1^Department of Endocrinology, Peking Union Medical College Hospital, Chinese Academy of Medical Sciences, Peking Union Medical College, Beijing 100730, China; ^2^Department of Health Management Center, Nanjing Drum Tower Hospital, The Affiliated Hospital of Nanjing University Medical School, Nanjing 210000, China

## Abstract

*Objective*: To investigate the effect of short-term recombinant human growth hormone (rhGH) replacement therapy on metabolic parameters in juvenile patients following craniopharyngioma (CP) resection. *Methods*. This retrospective study included 42 cases of juvenile patients that had undergone CP resection in the Department of Endocrinology at the Peking Union Medical College Hospital, from April 2013 to August 2020. According to whether they received growth hormone replacement therapy, the patients were divided into either the growth hormone replacement therapy (GHRT) group (30 cases) or the control group (12 cases). Changes in body mass index (BMI), BMI z-score, transaminase activity, fasting blood glucose (FBG) levels, blood lipid profile, and high-sensitivity C-reactive protein (hsCRP) levels were evaluated after one year of GHRT treatment. *Results*. The average age of the GHRT group was 13.00 (8.00–14.00) years old and these patients had undergone a CP operation an average of 2.00 (1.62–3.15) years earlier. Prior to receiving GHRT treatment, they received appropriate doses of adrenocortical hormone and thyroid hormone replacement therapy. After one year of GHRT treatment, the average BMI z-score decreased from 1.60 ± 1.76 to 1.13 ± 1.73 (*P*=0.005). Alanine aminotransferase (ALT) activity decreased from 26.50 (17.00∼98.00) U/L to 18.00 (13.00∼26.48) U/L (*P* ≤ 0.001), and similar changes were observed with regard to aspartate aminotransferase (AST) and glutamyl transferase (GGT) activity in the GHRT treatment group. The average total cholesterol (TC) decreased from 4.67 (4.10–6.14) mmol/L to 4.32 ± 0.85 mmol/L (*P*=0.002), and low-density lipoprotein (LDL) levels decreased from 3.05 ± 0.95 mmol/L to 2.56 ± 0.65 mmol/L (*P*=0.001) in the GHRT treatment group. The average blood urea nitrogen level decreased from 4.53 ± 1.09 mmol/L to 3.92 ± 0.82 mmol/L (*P*=0.016) and the average serum creatinine (SCr) level decreased from 55.59 ± 12.54 *µ*mol/L to 51.15 ± 10.51 *µ*mol/L (*P*=0.005) in the GHRT treatment group. The average hsCRP level decreased from 3.23 (1.79∼4.34) mg/L to 0.92 (0.42∼1.21) mg/L in the GHRT treatment group. In the control group, the average ALT activity increased from 26.58 ± 8.75 U/L to 42.58 ± 24.59 U/L (*P*=0.039), GGT activity increased from 19.0 (13.25–29.25) U/L to 25.00 (14.75–34.75) U/L (*P*=0.026), and LDL levels increased from 2.27 ± 0.76 mmol/L to 3.43 ± 1.28 mmol/L (*P*=0.04). *Conclusion*. GHRT treatment improves the metabolic parameters of juvenile patients that have undergone craniopharyngioma resection by reducing BMI z-scores, low-density lipoprotein, and hsCRP levels and improving liver function.

## 1. Introduction

Craniopharyngiomas (CPs) are rare embryonic malformations located in the sellar and parasellar areas. CPs are the most common nonneurogenic intracranial tumor in juvenile patients (<18 years old) [1], and account for 5–11% of all intracranial tumors in minors [2, 3]. The annual incidence rate of CPs is currently 0.5–2.5/million in children [1, 4, 5]. At present, tumor resection is the most optimal treatment [6].

After undergoing CP surgery, 50–80% of children will develop obesity [7–9]. The mechanism behind this phenomenon may be related to damage in the hypothalamus and pituitary caused either by the tumor itself or the operative procedure [10]. In addition, insufficient thyroid hormone supplementation, excessive glucocorticoid levels, and reduced daily activity are also important factors linked to the development of obesity [6]. Postoperative obesity increases the risk of metabolic syndrome and cardiovascular disease [11], and can lead to an increased incidence of sudden death and a reduction in the postoperative survival rate [12, 13]. Therefore, it is important to strictly control the metabolism of patients that have undergone CP surgery. 70–92% of CP patients suffered from postoperative growth hormone deficiency [14–16]. Some studies have shown that supplementation with growth hormone is helpful for supporting linear growth and healthy body composition [17–19]. However, there is still some controversy regarding the effects of GHRT treatment on metabolic indexes, such as body mass index (BMI) and BMI-SDS [18, 20, 21]. Therefore, this study aimed to clarify whether one year of GHRT treatment could improve BMI, the blood lipid profile, transaminase activity, and hsCRP levels in patients that have undergone CP surgeries.

## 2. Objects and Methods

This retrospective-designed study enrolled juvenile patients who visited and were followed up at the Department of Endocrinology at the Peking Union Medical College Hospital from April 2013 to August 2020. The patient inclusion criteria for the study included the following: the patient had received craniopharyngioma resection and had a definitive pathology; younger than 18 years old; rhGH was administered in the juvenile period and the treatment period lasted longer than one year; the control group was patients that did not receive any GHRT in the juvenile period. The patient exclusion criteria included the following: patients with other tumors; patients with other systemic diseases, such as SLE, chronic renal failure, and bone fracture; patients with poor treatment compliance or lacking follow-up information.

## 3. Methods

1. Clinical data were collected for all patients enrolled in the study, including age, gender, age at the time of the operation, height, weight, and the time and dosage for hormonal supplementation for multiple pituitary hormone deficiencies.
2. Biochemical indicators and hormone measurements analyzed: transaminase (ALT, AST, and GGT), blood lipids profiles (triglycerides, total cholesterol, high-density lipoprotein, and low-density lipoprotein), fasting blood glucose (FBG), blood urea nitrogen (BUN), serum creatinine (Scr), total cholinesterase (TC), lactate dehydrogenase (LDH), and high-sensitivity CRP (hsCRP).
3. GHRT: the risks and benefits of GHRT were assessed prior to growth hormone treatment, and informed consent was obtained from patients' parents. Patients were divided into two groups based on whether they received GHRT treatment: the GHRT group and the control group. In the GHRT group, 333∼1333 *µ*g/d of rhGH was administered, with the goal of improving IGF-1 levels to the age-matched normal range.
4. Primary outcomes: BMI (body mass index (kg/m^2^, BMI = weight/height [2]), height *z*-score and BMI z-score (according to age and gender, calculated using WHO Anthroplus software) [22]. Secondary outcomes: ALT, AST, GGT, TG, TC, HDL,LDL, FBG, BUN, Cr, cholinesterase(Che), LDH, and hsCRP measurements.

## 4. Statistical Analysis

**Figure 1:**
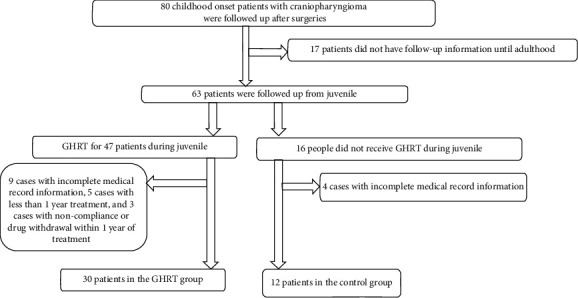
Flow chart of included patients.

**Figure 2:**
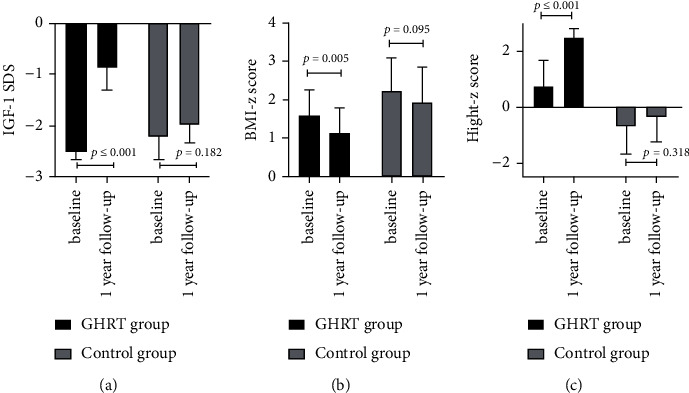
Change of IGF-1SDS, BMI z-score, height z-score in the GHRT group and control group.

**Figure 3:**
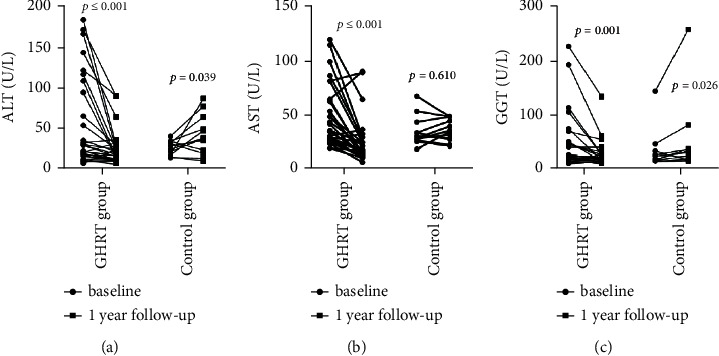
Decreased transaminase activity after one-year treatment with GHRT.

**Figure 4:**
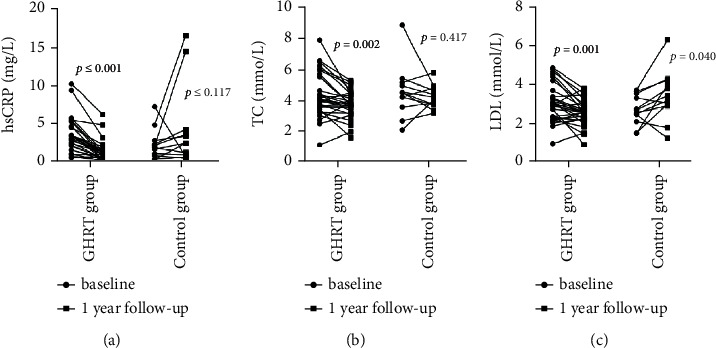
Decreased hsCRP, TC, and LDL.

**Figure 5:**
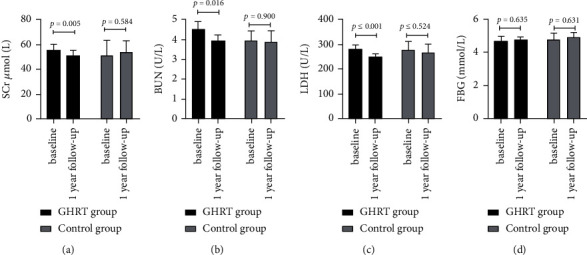
Changes in SCr, BUN, LDH, and FBG levels after one year of treatment.

Spss23.0 software was used for statistical analysis. The data were tested for normality, and the data that followed a normal distribution are expressed as mean ± SD. The data that did not follow a normal distribution are expressed as medians and quartile intervals, that is, median (P25–P75). The correlation of IGF-1SDS in the GHRT group with metabolic indexes was analyzed by linear regression analysis. Changes in metabolic indexes before and after treatment were compared using a paired *T* test, and the disparity between groups was determined using an independent sample *T* Test or a nonparametric test of independent samples. The enumeration data were calculated by frequency analysis, and groups were compared using *χ*^2^tests. *P* < 0.05 was considered statistically significant (see Figures 1–5). (see Tables 1 and 2).

## 5. Results

1. Baseline information: a total of 80 childhood-onset patients who had undergone craniopharyngioma surgery were followed up for this study in the Department of Endocrinology at the Peking Union Medical College Hospital. 30 patients (25 males and 5 females) were included in the GHRT group, and 12 patients (10 males and 2 females) were included in the control group (the patient flow chart for the study). The average ages of the treatment group and control group were 13.00 (8.00–14.00) years and 10.08 ± 3.42 years, respectively (*P*=0.241). The time that had passed since the operation was 2.00(1.62–3.15) and 1.80 (1.05–2.65) years for the treatment and control groups, respectively (*P*=0.354). Both groups were treated with appropriate doses of LT4, adrenocortical hormone, and desmopressin. The treatment group was treated with rhGH 19.98 ± 9.99 *µ*g/kg/d (1U = 333 *µ*g).
2. Changes in IGF-1 SDS BMI, BMI z-score, and height z-score after one year of GHRT treatment
3. After one year of rhGH treatment, the average IGF-1 SDS of the GHRT group increased from −2.59 (−2.81∼2.37) to −1.26 (−1.70∼0.18); the average BMI of the GHRT group decreased from 22.61 ± 5.51 kg/m^2^ to 22.14 ± 5.23 kg/m^2^ (*P*=0.217), the average height z-score of the treatment group increased from −1.98 ± 1.70 to −1.21 ± 1.45 (*P* ≤ 0.001), and the average BMI z- score decreased from 1.60 ± 1.76 to 1.13 ± 1.73 kg/m^2^(*P*=0.005). No significant changes in the average IGF-1 SDS, BMI, height z-score, and BMI z- score was observed for the control group during the one-year follow-up period (*P*=0.182, *P*=0.318, *P*=0.673, and *P*=0.095, respectively). The average change in the BMI for the GHRT and control groups was—0.47 ± 2.05 and 0.25 ± 1.99 kg/m^2^, *P*=0.350, respectively. The average change in BMI z-scores for the GHRT and control groups was −0.47 ± 0.84vs. −0.32 ± 0.60 (*P*=0.588), respectively. The average change in height z-score for the GHRT and control groups was 0.56(0.21∼1.37) vs. 0.31 ± 1.03 (*P*=0.089), respectively.
4. Decreased transaminase activity after one-year treatment with GHRT
5. After undergoing one year of rhGH replacement treatment, alanine aminotransferase (ALT) activity levels in the GHRT group decreased from 26.50(17.00–98.00) U/L to 18.00 (13.00–26.48) U/L (*P* ≤ 0.001).Aspartate aminotransferase (AST) activity levels in the GHRT group decreased from 36.00 (28.00–65.00) U/L to 29.40 (23.00–35.00) U/L (*P* ≤ 0.001), and GGT activity levels decreased from 22.00 (14.50–45.00) U/L to 18.50 (12.75–24.54) U/L (*P* ≤ 0.001). After one year of follow-up, the average ALT activity level in the control group increased from 26.58 ± 8.75 U/L to 42.58 ± 24.59 U/L, and the average GGT activity increased from 19.00 (13.25–29.25) U/L to 25.0 (14.75–34.75) U/L (*P*=0.026) When compared to the control group, the average AST, ALT, and GGT activity levels in the GHRT group were significantly decreased (all *P* < 0.05). Details are in Table 2.
6. Decreased TC, LDL, and hsCRP
7. The average TC in the GHRT group decreased by 0.65 ± 1.01 mmol/L (*P*=0.002), and no significant change was observed in the control group after one year of treatment (*P*=0.417). HDL levels did not significantly change in either group (*P*=0.574, *P*=0.073, respectively). In the GHRT group, the average LDL level decreased from 3.05 ± 0.95 to 2.56 ± 0.65 mmol/L (*P*=0.001), while in the control group, the average LDL level decreased from 2.27 ± 0.76 to 3.43 ± 1.28 mmol/L (*P*=0.040). The average hsCRP level decreased by 1.93 (2.47∼0.97) mg/L for the GHRT treatment group and increased by 0.64 (−0.28∼1.99) mg/L for the control group (*P* ≤ 0.001 when comparing the two treatment groups). Details are in Table 2.
8. Changes in FBG, SCr, BUN, and LDH levels after one year of treatment
9. There was no significant change in FBG levels in either treatment group. After one year of follow-up, the average serum creatinine level in the GHRT group decreased from 55.59 ± 12.54 to 51.15 ± 10.51 *µ*mol/L (*P* ≤ 0.005) and increased in the control group from 51.25 ± 19.08 to 53.92 ± 13.66 *µ*mol/L (*P*=0.584). The average urea nitrogen level in the GHRT group decreased from 4.53 ± 1.09 to 3.92 ± 0.82 mmol/L (*P*=0.016) and there was no change observed in the control group. The average LDH activity level decreased from 281.14 ± 43.03 to 250.23 ± 32.85 U/Lfor the GHRT group. Details are in Table 2. The IGF-SDS increase in the GHRT group only negatively correlated with the change of BUN (*β* (95% CI) P value = −0.53 (−1.00, −0.06) 0.036), but not other indexes.
10. Tumor recurrence and other reported side effects: One patient in the GHRT group was found to have tumor recurrence, so an operation was carried out to resect the tumor. No tumor recurrence was detected in the control group. Side effects from treatment such as edema, headache, and skin rash were not reported by any patients.

## 6. Discussion

Management of metabolism is particularly important for juvenile patients that undergo a CP resection [6]. Poor metabolic parameters decrease the quality of life [8], increase the risks of cardiovascular and cerebrovascular diseases, and reduce the long-term survival rate of CP resection patients [23]. Our study found that short-term (-one year) treatment with rhGH can reduce patient BMI z-scores, improve liver function, reduce TC levels, LDL, hsCRP levels, SCr, BUN, and LDH levels, without increasing fasting blood glucose levels. These findings indicate that rhGH treatment can ameliorate risk factors for cardiovascular disease in juvenile patients that have undergone CP surgery. Supplementary Table 1.

In this study, 73.8% (31/42) of the children were overweight or obese (BMI z- score > 1), far exceeding the overall incidence of obesity in Chinese children (6.2%) [24]. After one year of GHRT treatment, the average BMI z-score in the treatment group decreased from 1.60 to 1.13 (*P*=0.005). This may be related to growth hormone-induced lipolysis. Growth hormone treatment can induce and activate the MEK-ERK pathway to phosphorylate PPAR-*γ*, which results in decreased expression of FSP27 and subsequently enhances lipolysis [25]. Evidence has shown that long-term supplementation with growth hormone (666 *µ*g/m^2^), when compared with placebo, can achieve sustained lipolysis and fat mass loss, ultimately helping attain normal body composition in adults with growth hormone deficiency [26]. However, the effect of rhGH on the BMI of patients with CP is still controversial. Schoenle reported that one year of GHRT treatment decreases BMI-SDS in prepubertal children that have undergone CP surgery [27]. Previous studies by our group have also found that 4–6 months of GHRT treatment improves the body composition and metabolic status of adult patients [17]. However, some studies found that the beneficial effect of GHRT treatment on BMI in patients with CP is very slight after long-term GHRT treatment [20, 28]. In our study, while the average BMI z-score decreased after the use of rhGH, there were no significant changes between the GHRT and control groups (*P*=0.588). Due to the concerns about tumor recurrence, we used a low dose of rhGH. After treatment, the average IGF-1 level increased by −1.26 (−1.70∼0.18) SD, which was lower than the age-matched mean value. This finding may explain why the decrease in BMI z- score is not so obvious. More studies with a larger sample size could help address this point. In recent years, a compilation of existing studies demonstrated that the application of appropriate growth hormone doses does not increase the risk of tumor recurrence [6, 29]. Therefore, with a prudent and higher dosage of rhGH, the beneficial effect of treatment on BMI would likely be more significant.

We found that following GHRT treatment, the level of transaminase activity decreased dramatically. The incidence of nonalcoholic fatty liver disease (NAFLD) and nonalcoholic steatohepatitis (NASH) in adults with growth hormone deficiency have been reported to be 70% and 21%, respectively, which are much higher than in the normal population (12% and 5%, respectively) [30, 31]. Accordingly, GHRT treatment has been found to effectively alleviate hepatic steatosis, fibrosis, and inflammation in patients with GH deficiency combined with NASH or NAFLD [31, 32]. Our findings directly reflect the beneficial effect of rhGH treatment on the incidence of fatty liver in children. The protective mechanism for GH in the liver includes inhibition of hepatic fat synthesis [33], inhibition of Kupffer cell function (34), reduction of hepatocyte oxidative stress [34], induction of Kupffer cell senescence [35], promotion of hepatocyte proliferation [36], and induction of autophagy [37]. Long-term GH deficiency may cause hepatopulmonary syndrome due to the development of obesity, and GHRT supplementation may alleviate symptoms by improving liver fibrosis and cirrhosis [38]. Our study found that the lack of growth hormone in the control group led to the deterioration of transaminase indicators, indicating that it is critical to use rhGH to protect the liver from fibrosis and cirrhosis.

Previous studies have shown that GHRTsupplementation for one year improves the blood lipid profile, including lowering TC, TG, and LDL levels, and increasing HDL levels [39, 40]. Our study found that growth hormone treatment may lower TC and LDL levels, but has little effect on the increase in HDL levels and the decrease in TG levels. This may be due to the relatively small patient sample size. Our study also found that GHRT treatment can reduce hsCRP levels, which is a critical indicator of cardiovascular disease and general inflammation status [41]. Consistent with these results, recent studies have demonstrated that rhGH has antiinflammatory effects [42] and may improve the metabolic status of obese patients by inhibiting inflammatory factors and promoting lipolysis.

The study also found that GHRT treatment did not alterFBG levels within a year of CP surgery. On one hand, rhGH can increase the concentration of free fatty acids, induce insulin resistance, and increase fasting blood glucose levels [43]. On the other hand, many studies have confirmed that long-term low-dose growth hormone therapy can reduce visceral fat accumulation and optimize body composition, thus improving the sensitivity of insulin action [44–46]. In our study, with a relatively low dosage of GH supplementation, IGF-1 levels were maintained in the normal low range, wh./ich further confirmed that a physiological dose of GH had no significant detrimental effect on glucose metabolism.

During the follow-up period, we found that GHRT treatment may improve serum creatinine, BUN, and LDH levels and activity. Decreased creatinine levels may reflect the promotion of muscle synthesis and reduction of muscle decomposition [43, 47]. Furthermore, increasing renal blood flow and glomerular filtration rateviarhGH treatment may also help lower creatinine levels [48–52]. However, there are two sides to the effect of growth hormone treatment on the kidneys. Patients with acromegaly are prone to proteinuria [52]. In the normal healthy population, subcutaneous supplementation of IGF-1 can also lead to elevated urinary protein levels [48]. Therefore, the effect of growth hormone on kidney function should be reevaluated by studies with larger sample sizes and longer follow-up periods. Our study found that BUN levels decreased after GHRT treatment, which may be related to the effect of growth hormone on protein synthesis [43, 53]. To our surprise, we found that LDH levels were decreased, a finding that has not been reported in previous studies. Although the clinical significance of this finding is unknown, this change may be related to the positive effects of growth hormone supplementation on heart, kidney, liver, and muscle function and health.

Some limitations to this study should be addressed. First, as a retrospective study, selective bias may exist. For example, there is no randomization in selecting patients for the GHRT treatment or control groups. Second, the metabolic effect of GHRT treatment was mainly evaluated via analysis of serum biochemistry, not by more accurate MR imaging and pathological examination. Third, the follow-up time should be extended. Finally, the relationship between the dose of growth hormone and the therapeutic effect was not further analyzed in this study due to the small sample size.

In conclusion, GHRT treatment can reduce BMI z-scores, improve liver function, blood lipid profiles, and hsCRP levels, and reduce serum creatinine and BUN levels in children following a CP resection operation. These beneficial metabolic changes observed with GHRT treatment could improve patients' quality of life, lower the risk of cardiovascular and hepatic disease, and increase overall survival.

## Figures and Tables

**Table 1:** Comparison of the GHRT and control group baseline characteristics.

Characteristic	GHRT group (*n* = 30)	Control group (*n* = 12)	*P*
Gender (male/female, *n*)	25/5	10/2	>0.999
Age (year)	13.00 (8.00∼14.00)	10.08 ± 3.42	0.241
Age at time of surgery (year)	9.50 (5.38∼11.53)	7.77 ± 3.46	0.477
Time sincere section (year)	2.00 (1.62∼3.15)	1.8(1.05∼2.65)	0.354
LT4 dosage (*µ*g/d/m^2^)	45.84 ± 12.92 (*n* = 28)	49.24 ± 22.45 (*n* = 10)	0.660
Hydrocortisone dosage (mg/d/m^2^)	10.10 ± 5.23 (*n* = 27)	7.26 ± 6.05 (*n* = 11)	0.156
Sex hormone supplement (*n*)	11	1	0.128
rhGH dosage (*µ*g/kg/d)	19.98 ± 9.99	0	0.001
Desmopressin (*n*)	28	12	>0.999
Overweight or obese (*n*) (BMI-Z >1)	20 (66.7%)	11 (91.7%)	0.133
IGF-1 <—2sd (*n*)	25	8	0.406

**Table 2:** Changes in metabolic parameters for patients with craniopharyngioma after rhGH replacement therapy.

Metabolic parameter	GHRT group (*n* = 30)			Control group (*n* = 12)			Change after one year follow-up		
	Before	1-year GHRT	*P*	Baseline	1-year follow-up	*P*	Change for GHRT group	Change for the control group	*P*
BMI (kg/m^2^)	22.61 ± 5.51	22.14 ± 5.23	0.217	23.78 ± 5.50	24.03 ± 5.82	0.673	−0.47 ± 2.05	0.25 ± 1.99	0.350
BMI z-score	1.60 ± 1.76	1.13 ± 1.73	0.005	2.23 ± 1.37	1.91 ± 1.48	0.095	−0.47 ± 0.84	−0.32 ± 0.60	0.588
Height z-score	−1.98 ± 1.70	−1.21 ± 1.45	≤ 0.001	−0.66 ± 1.61	−0.35 ± 1.36	0.318	0.56 (0.21∼1.37)	0.31 ± 1.03	0.089
IGF-1 (SD)	−2.59 (−2.81∼−2.37)	−1.26 (−1.70∼0.18)	≤ 0.001	−2.57 (−2.61∼−1.57)	−1.99 ± 0.56	0.182	1.36 (1.04∼2.30)	0.25 ± 0.53	≤ 0.001
ALT (U/L)	26.50 (17.00∼98.00)	18.00 (13.0∼26.48)	≤ 0.001	26.58 ± 8.75	42.58 ± 24.59	0.039	−7.00 (−32.5∼−2.0)	16.00 ± 23.62	0.001
AST (U/L)	36.00 (28.00∼65.00)	29.40 (23.00∼35.00)	≤ 0.001	30.00 (27.25∼41.50)	36.42 ± 9.99	0.610	−7.50 (−43.50∼−9.00)	1.17 ± 9.71	0.007
GGT (U/L)	22.00 (14.50∼45.00)	18.50 (12.75∼24.54)	0.001	19.00 (13.25∼29.25)	25.0 (14.75∼34.75)	0.026	−5.00(−20.34∼0.25)	7.00 (1∼19.5)	≤ 0.001
TG (mmol/L)	1.45 (0.71∼2.02)	1.34 (0.81∼1.83)	0.551	1.52 (0.91∼3.44)	1.53 ± 0.60	0.388	−0.26 ± 1.40	−0.33 (−1.03∼0.55)	0.540
TC (mmol/L)	4.67 (4.10∼6.14)	4.32 ± 0.85	0.002	5.14 ± 1.66	4.80 ± 0.74	0.417	−0.65 ± 1.01	−0.34 ± 1.41	0.436
HDL (mmol/L)	1.06 (0.76∼1.42)	1.12 ± 0.33	0.574	0.95 ± 0.27	1.06 ± 0.28	0.073	0.02 ± 0.40	0.10 ± 0.18	0.513
LDL (mmol/L)	3.05 ± 0.95	2.56 ± 0.65	0.001	2.27 ± 0.76	3.43 ± 1.28	0.040	−0.49 ± 0.72	0.74 ± 1.10	≤0.001
FBG (mmol/L)	4.72 ± 0.73	4.77 ± 0.38	0.635	4.87 ± 0.49	4.92 ± 0.49	0.631	0.06 ± 0.66	0.05 ± 0.35	0.971
BUN (mmol/L)	4.53 ± 1.09	3.92 ± 0.82	0.016	3.90 ± 0.86	3.94 ± 0.82	0.900	−0.61 ± 1.30	0.04 ± 1.10	0.137
SCr (*µ*mol/L)	55.59 ± 12.54	51.15 ± 10.51	0.005	51.25 ± 19.08	53.92 ± 13.66	0.584	−4.43 ± 8.06	5.00 (4.00∼15.0)	0.004
Che (KU/L)	10.39 ± 2.16	10.6 (9.7∼11.8)	0.136	9.97 ± 1.05	10.04 ± 1.14	0.627	0.39 ± 1.94	0.16 ± 1.13	0.707
LDH (U/L)	281.14 ± 43.03	250.23 ± 32.85	≤ 0.001	278.00 ± 53.16	269.73 ± 50.26	0.524	−31.21 ± 40.71	−8.27 ± 43.59	0.114
hsCRP (mg/L)	3.23 (1.79∼4.34)	0.92 (0.42∼1.21)	≤ 0.001	1.83 (0.79 ± 2.52)	2.26 (0.63∼3.91)	0.117	−1.93 (−2.47∼−0.97)	0.64 (−0.28∼1.99)	≤ 0.001

## Data Availability

All data that support the findings of this study are available from the corresponding author on reasonable request.

## Supplementary Materials

Supplementary Materials Table 1: the correlation of IGF-SD increasing in the GHRT group with metabolic parameters.
