# Supplementary material for: Metabolic Effects of Recombinant Human Growth Hormone Replacement Therapy on Juvenile Patients after Craniopharyngioma Resection
Source: Int J Endocrinol. 2022 Jul 6;2022:7154907. doi: 10.1155/2022/7154907 (PMC9279072; doi:10.1155/2022/7154907)
Supplement: Supplementary Materials — Table 1: the correlation of IGF-SD increasing in the GHRT group with metabolic parameters. [file 7154907.f1.docx]

Supplementary materials table 1: The correlation of IGF-SD increasing in the GHRT group with metabolic parameters.

| Metabolic parameters | β (95%CI) | P-value |
| --- | --- | --- |
| ALT(U/L) | 3.30 (-14.44, 21.04) | 0.718 |
| AST(U/L) | 4.41 (-5.18, 14.00) | 0.375 |
| GGT(U/L) | -1.63 (-15.30, 12.05) | 0.818 |
| TG (mmol/L) | -0.51 (-1.02, 0.01) | 0.065 |
| TC (mmol/L) | -0.07 (-0.47, 0.33) | 0.731 |
| HDL (mmol/L) | 0.13 (-0.72, 0.97) | 0.771 |
| LDL (mmol/L) | 0.02 (-0.26, 0.31) | 0.869 |
| FBG (mmol/L) | -0.00 (-0.26, 0.26) | 0.996 |
| BUN (mmol/L) | -0.53 (-1.00, -0.06) | 0.036 |
| Che(KU/L) | -0.55 (-1.29, 0.18) | 0.151 |
| Scr (µmol/L) | -0.17 (-3.34, 2.99) | 0.915 |
| hsCRP(mg/L) | 0.13 (-0.72, 0.97) | 0.771 |
